# Supplementary material for: Temporal chromatin accessibility changes define transcriptional states essential for osteosarcoma metastasis
Source: Nat Commun. 2023 Nov 8;14:7209. doi: 10.1038/s41467-023-42656-x (PMC10632377; doi:10.1038/s41467-023-42656-x)
Supplement: Supplementary file 1 — Supplemental Information [file 41467_2023_42656_MOESM1_ESM.pdf]

Patient SJ ID#	Age	Gender	Status	Site	Location	Treated	Germline Mutation	Common mutations
SJOS001107_D1	13 yr	Male	Unknown	Primary	Humerus	No	No	TP53, DLG2, STX8, RNF111
SJOS030589_D1	6yr	Female	Recurrent	Primary	Femur	Yes	No	WRNIP1
SJOS031478_D4	15 yr	Male	Diagnostic	Primary	Humerus	Yes	No	none
SJOS001107_M2	14 yr	Male	Recurrent	Metastatic	Lung	No	No	TP53, DLG2, STX8
SJOS013768_M1	15 yr	Female	Recurrent	Metastatic	Lung	Yes	No	DLG2, ARTX, WRNIP1, AFF3, IL12RB1
SJOS030589_M3	6yr	Female	Recurrent	Metastatic	Lung	Yes	No	WRNIP1
SJOS030605_R2	10 yr	Female	Recurrent	Metastatic	Chest wall	yes	No	TP53
SJOS030645_D1	8 yr	Male	Recurrent	Metastatic	Lung	No	No	TP53, STX8
SJOS031478_D2	17 yr	Male	Recurrent	Metastatic	Lung	No	No	RB1

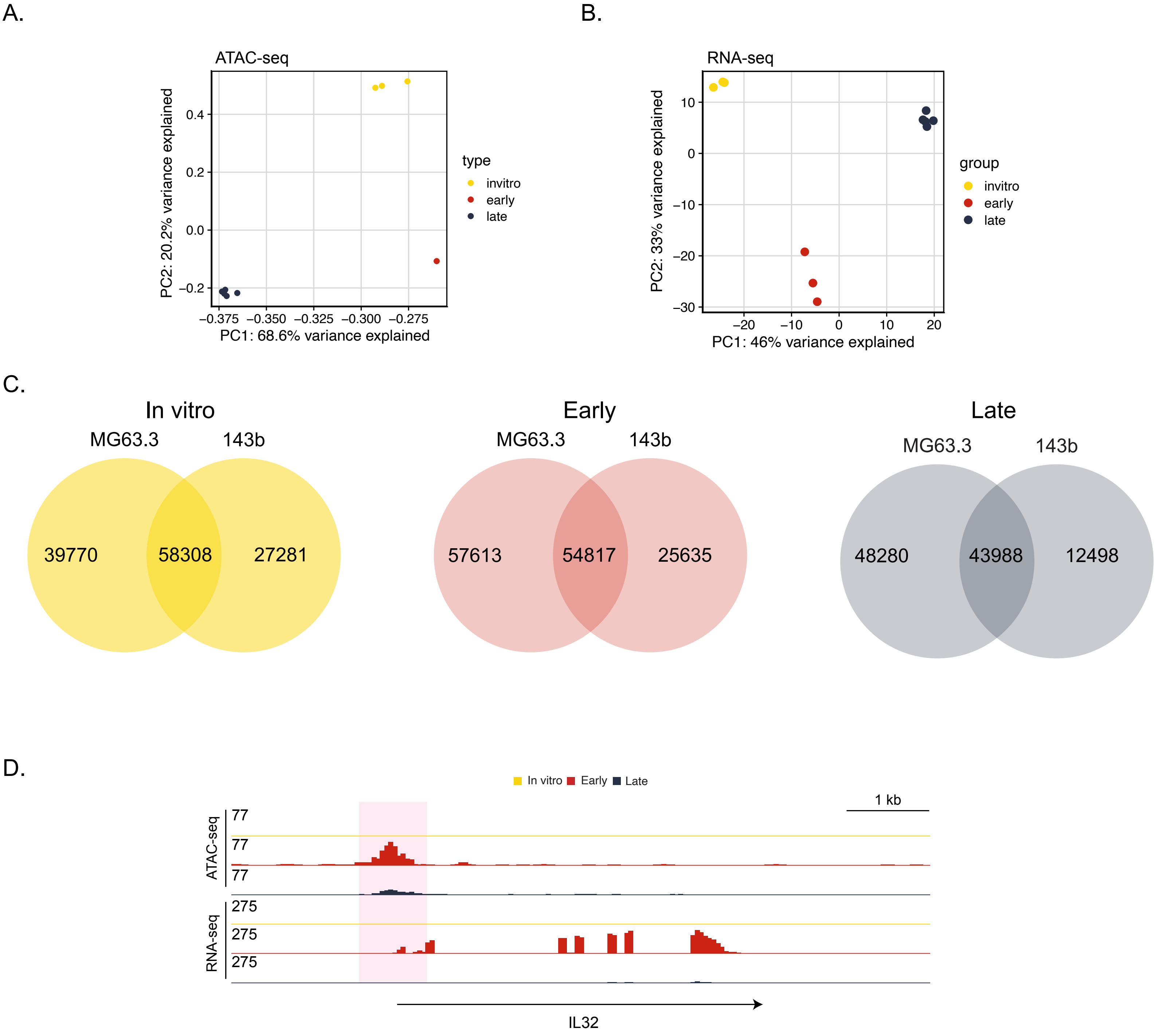

Supp Fig 1: Profiling of chromatin accessibility and gene expression in 143b-HOS during metastasis. A) Principal component analysis of open chromatin across the three conditions. Each point represents a distinct biological replicate. **B)** Principal component analysis of gene expression across the three conditions. **C)** Overlap between ATAC-seq data sets from cell lines profiled. Number of peaks in each set are displayed in the venn diagram. **D)** Genome browser view of an early-specific change in chromatin accessbility with corresponding increases in gene expression.

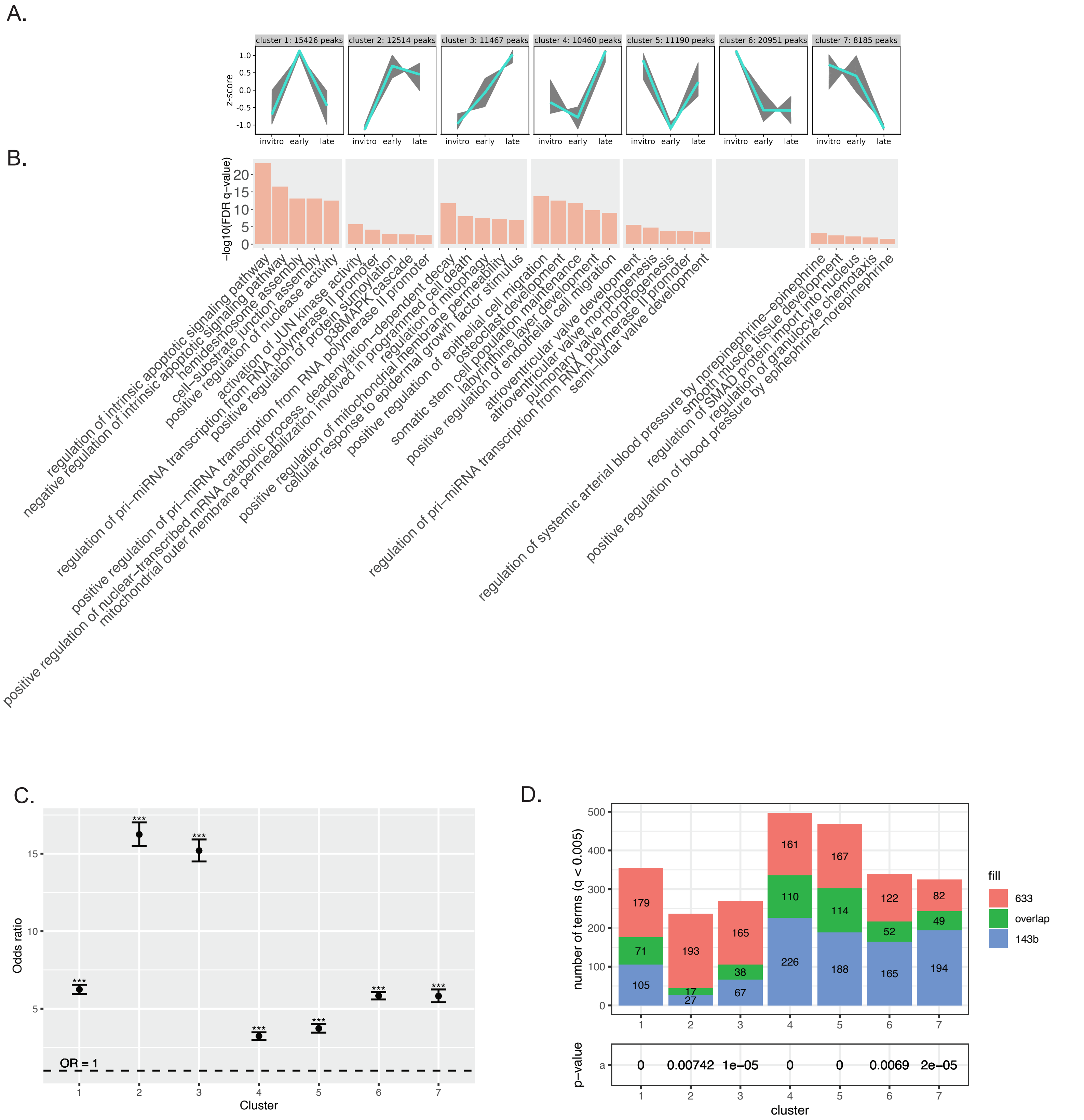

Supp Fig 2: Identification of dynamic clusters of accessible chromatin in 143b-HOS. A) Clusters of dynamic accessible regions identified in the 143b cell line based on k-means clustering. The teal line in the middle represents the mean change in accesibility for all peaks within a given cluster. **B)** Peak ontology of each cluster based on GREAT. Top 5 terms for each cluster are shown. **C)** Significance of overlapping accessible regions between analagous clusters in MG63.3 and 143b. Data displayed are odds ratio with confidence intervals. P-values were calculated using one-sided Fisher's test (* = p < 0.05, ** = p < 0.005, *** = p < 0.0005). Dot represents the odds ratio, while the error bars signify the confidence interval. **D)** Signifiance of overlap between significant GREAT terms for each cell line's dynamic cluster. P-values were calculated using a one-sided hypergeomtric test.

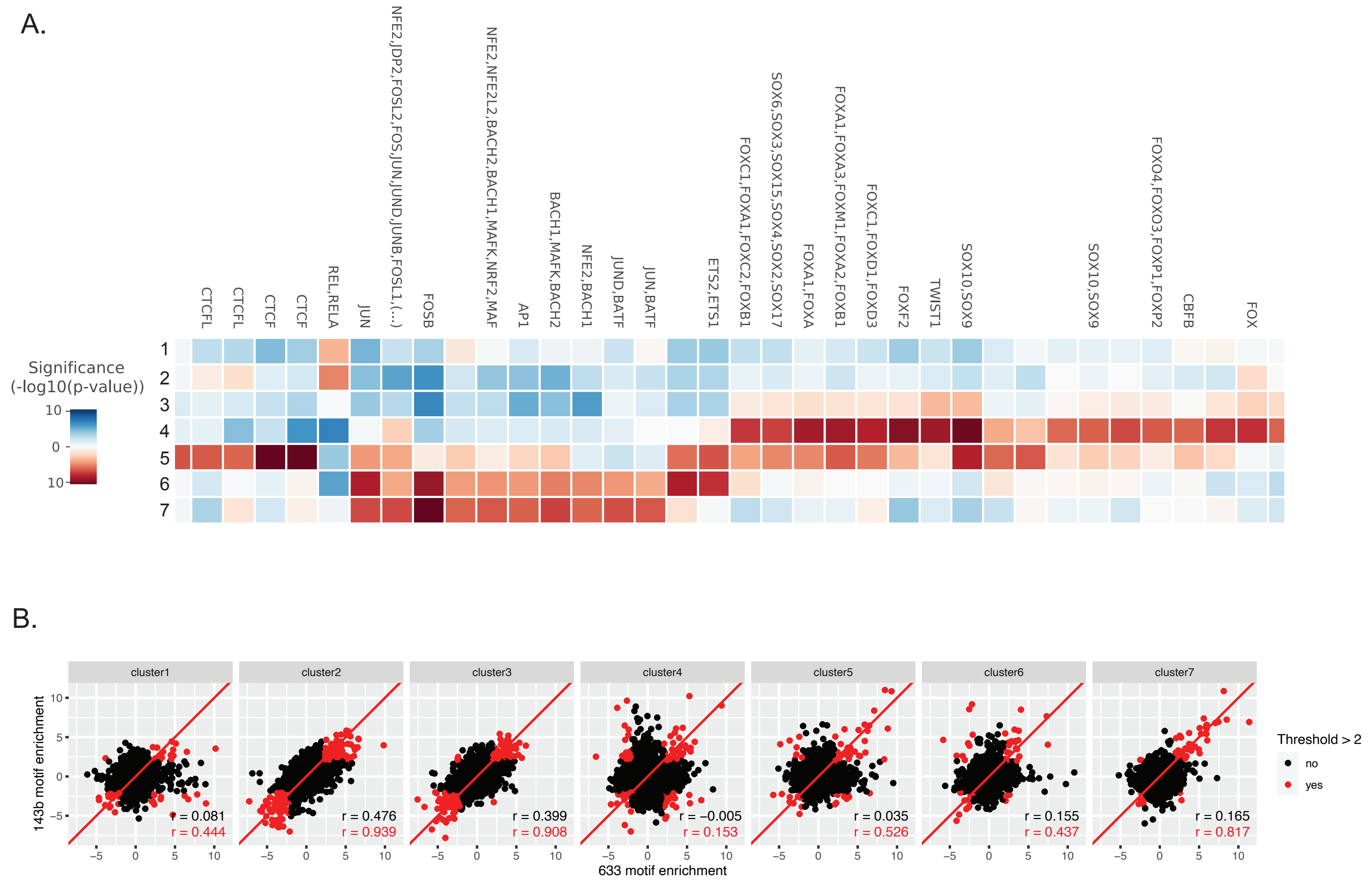

Supp Fig 3: Overlap of cluster-enriched motifs between two osteosarcoma cell lines shows convergent biology. A) Differential motif enrichment for peaks within each dynamic cluster. **B)** Correlation between enriched motifs for dynamic clusters in MG63.3 and 143b. Motifs highlighted in red reach a significance threshold of abs(-log10(p)) > 2 for either cell line.

A.

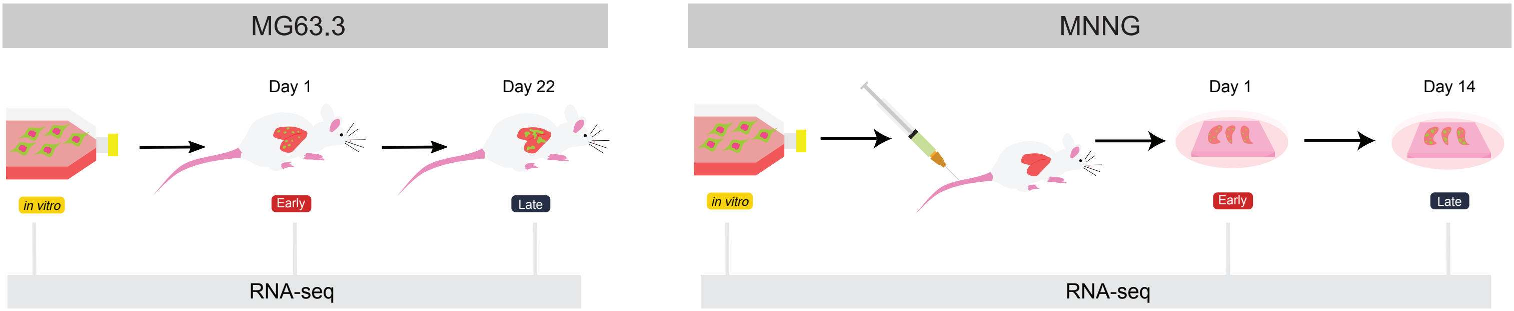

B.

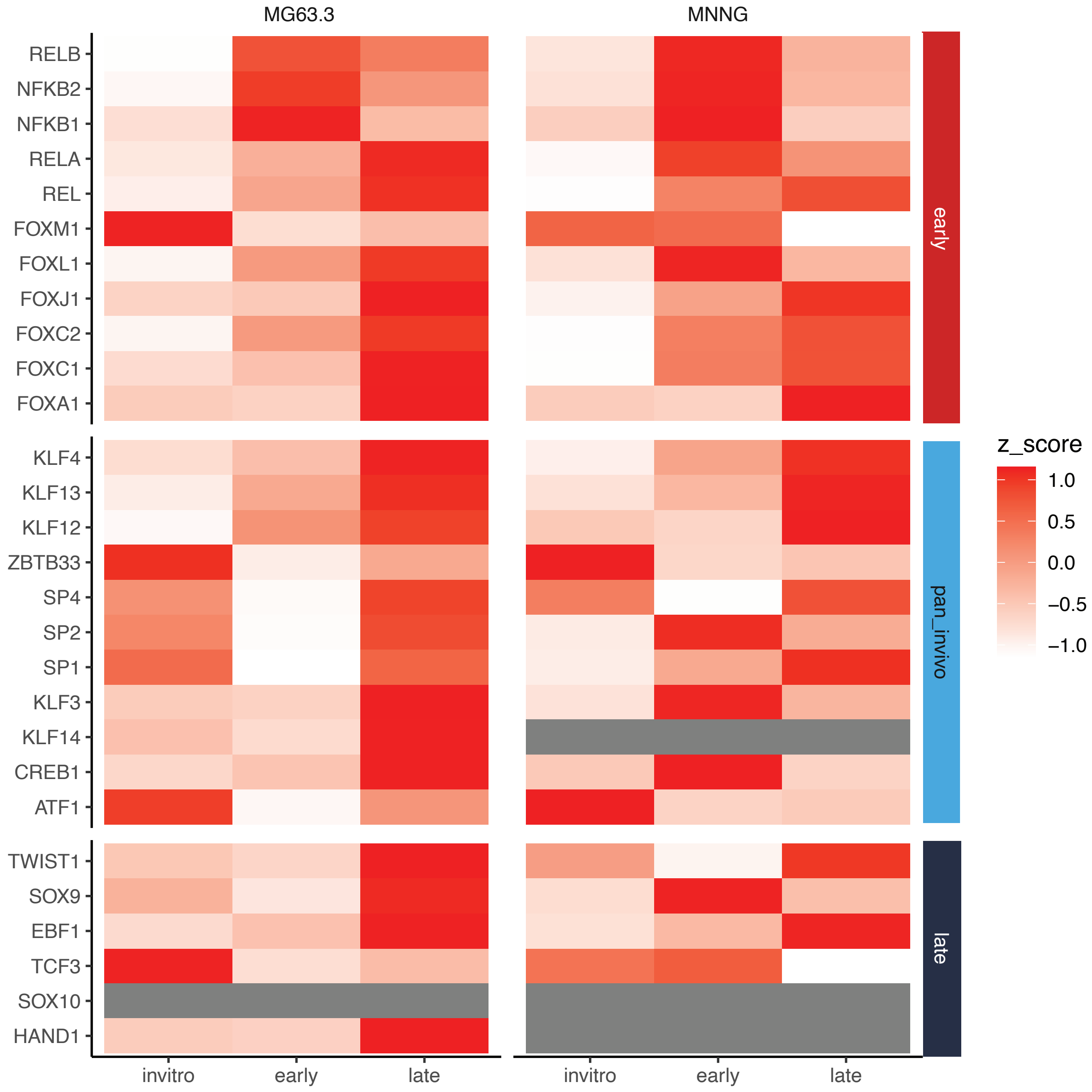

Supp Fig 4: Cluster regulators show similar expression dynamics across multiple metastatic osteosarcoma cell lines. A) Schematic comparing RNA-seq profiling experiments of MG63.3 and MNNG cell lines. MG63.3 early and late conditions were isolated from a fully *in vivo* model of metastasis, whereas MNNG cells were isolated from the *ex vivo* PuMA model. **B)** Row-normalized heatmap displaying trends in expression for all putative cluster-regulating TFs in MG63.3 and MNNG.

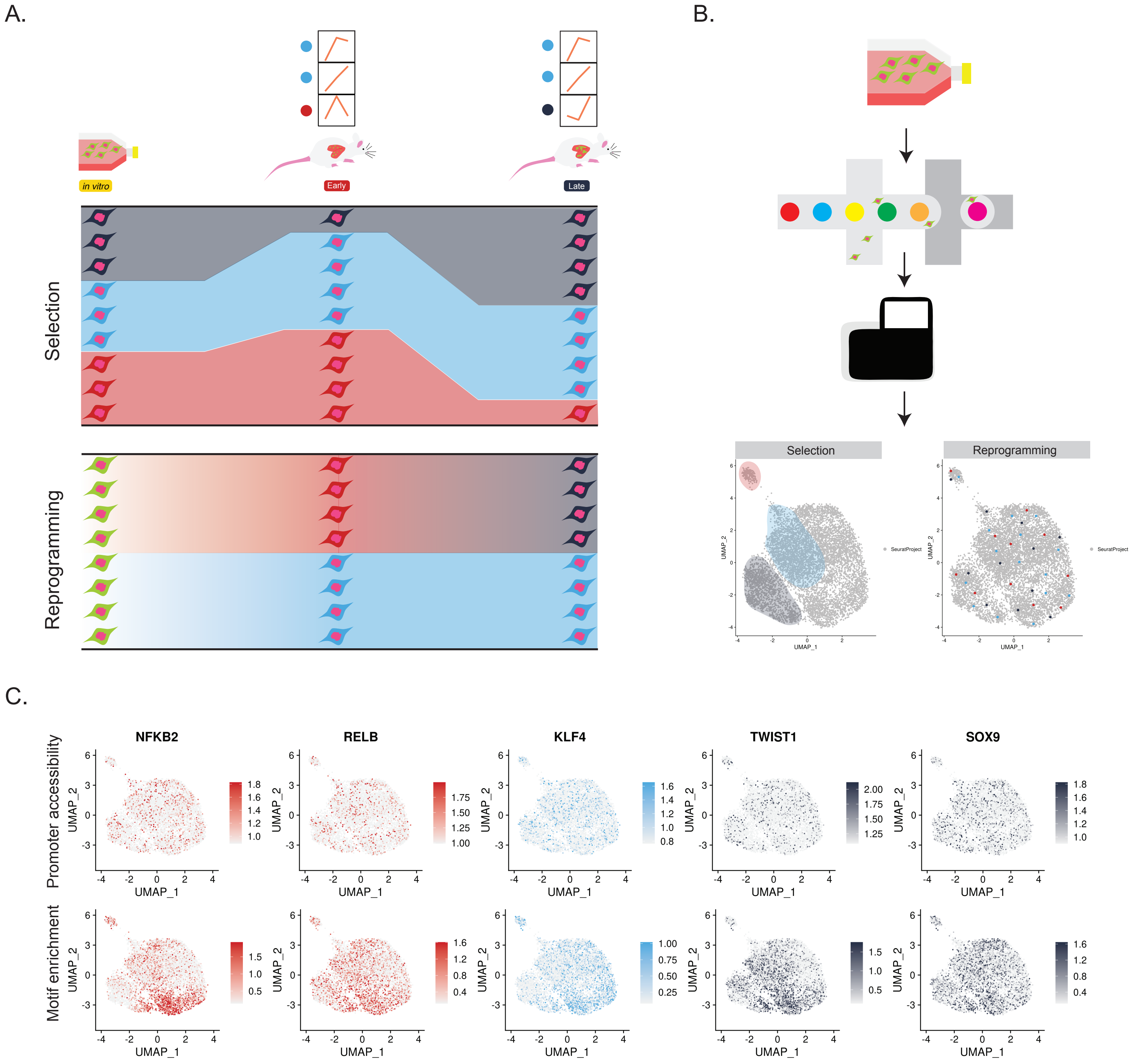

Supp Fig 5: Dynamic clusters of accessible chromatin are not due to subclonal selection *in vivo*. A) Schematic of potential causes of dynamic ATAC-seq peaks observed *in vivo*. **B)** Diagram illustrating single cell-ATAC seq experiment and expected distribution of marker transcription factors depending on selection or reprogramming. **C)** Single cell promoter accessibility and motif enrichment for putative dynamic transcription factors.

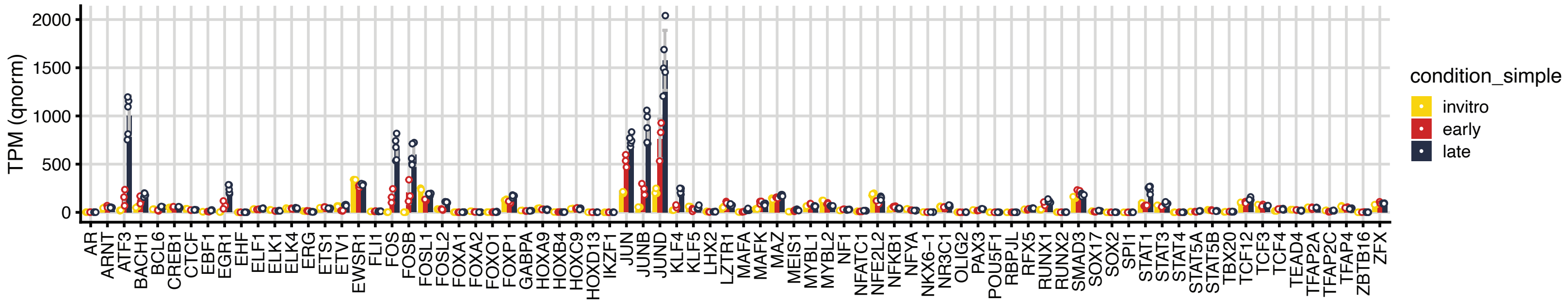

Supp Fig 6: Relative expression of screen TFs over the metastasis time course. RNA expression of all 78 TFs present in the CRISPR screen library. Barplot shows the average TPM (quantile-normalized) across all replicates for each time point, while individual points represnet TPM for each replicate. Data are displayed as mean +/- SD, with n = 3 *in vitro*, n = 3 early, n = 5 late.

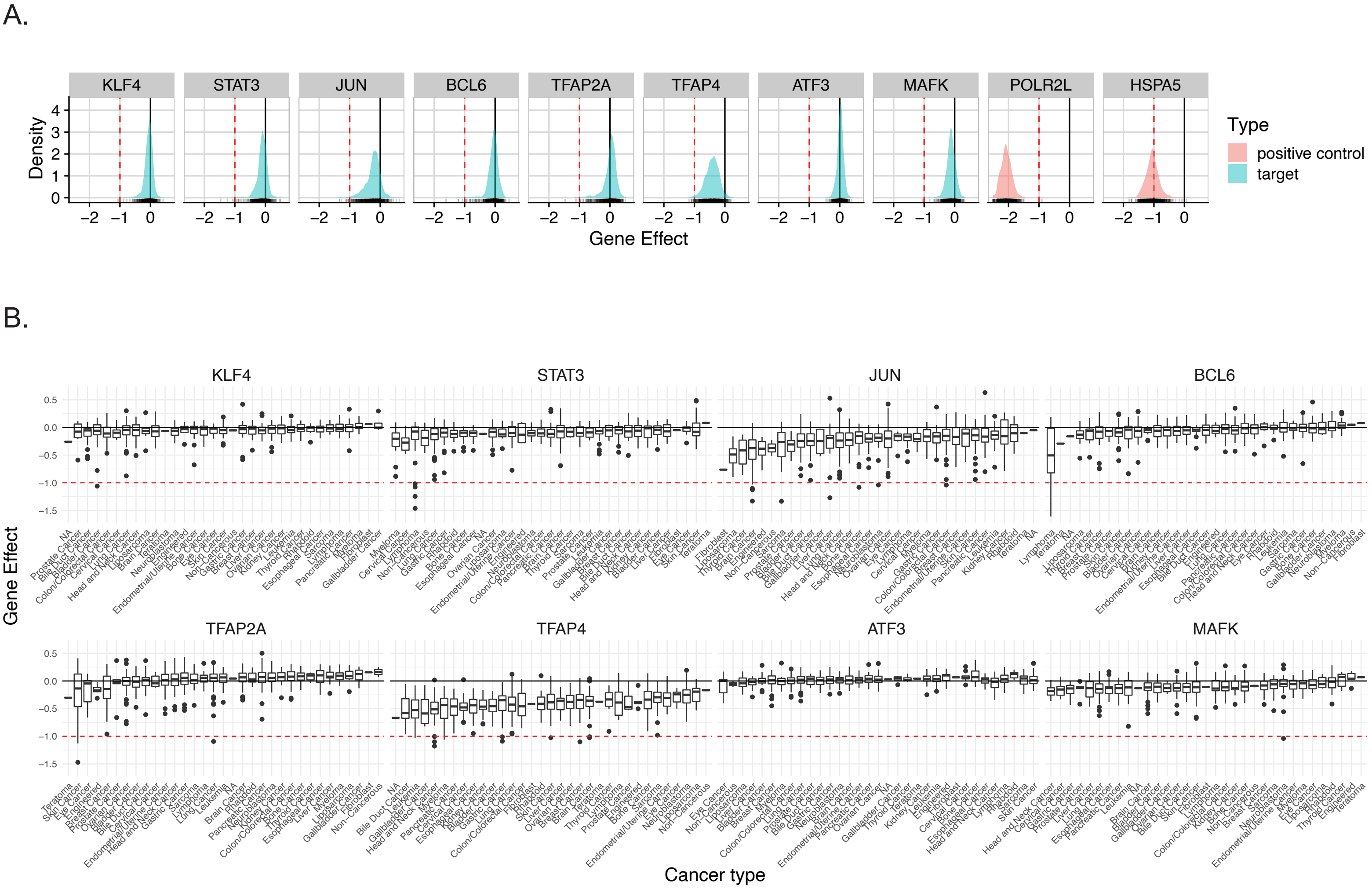

Supp Fig 7: DepMap essentiality data demonstrates specificity of metastasis-dependency genes. A) Distribution of gene effect for each *in vivo* hit across all cell lines included in the DepMap database. Genes with an effect less than -1 are dependency genes. **B)** Boxplots highlighting lineage-specific gene effect for each *in vivo* hit. The number of samples represented by each boxplot are as follows: Bile Duct Cancer = 29, Bladder Cancer = 29, Bone Cancer = 38, Brain Cancer = 78, Breast Cancer = 43, Cervical Cancer = 13, Colon/Colorectal Cancer = 53, Endometrial/Uterine Cancer = 32, Engineered = 5, Esophageal Cancer = 31, Eye Cancer = 7, Fibroblast = 1, Gallbladder Cancer = 4, Gastric Cancer = 34, Head and Neck Cancer = 61, Kidney Cancer = 26, Leukemia = 49, Liposarcoma = 8, Liver Cancer = 22, Lung Cancer = 125, Lymphoma = 29, Myeloma = 21, NA = 1, Neuroblastoma = 31, Non-Cancerous = 2, Ovarian Cancer = 57, Pancreatic Cancer = 44, Prostate Cancer = 6, Rhabdoid = 13, Sarcoma = 24, Skin Cancer = 65, Teratoma = 1, Thyroid Cancer = 8. Boxplots represent the interquartile range where the top of the box is third quartile, the bottom of the box is first quartile, and the midline is median. Whiskers extend to 1.5 times IQR and dots represent outliers.

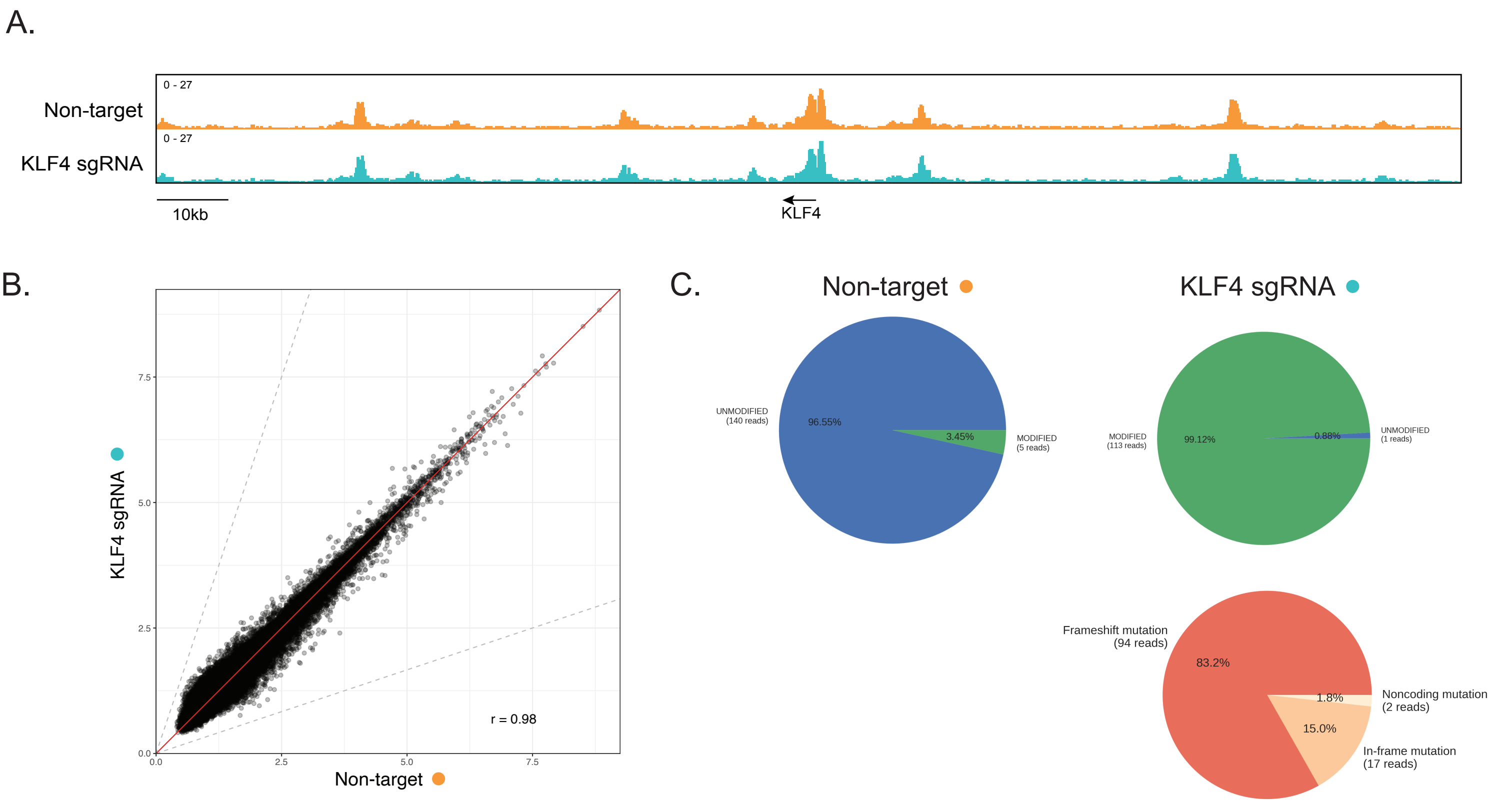

Supp Fig 8: *KLF4* knockout does not affect the *in vitro* enhancer landscape of MG63.3 cells. A) Genome browser view of H3K27ac signal at the *KLF4* locus in *KLF4* knockout cells and non-target control transduced cells. **B)** Comparison of genome-wide H3K27ac ChIP-seq signal (RPKM) between *KLF4* knockout cells and non-target control transduced cells. **C)** Summary of *KLF4* editing for both cell lines based on H3K27ac ChIP-seq reads.
