# Supplementary material for: Temporal chromatin accessibility changes define transcriptional states essential for osteosarcoma metastasis
Source: Nat Commun. 2023 Nov 8;14:7209. doi: 10.1038/s41467-023-42656-x (PMC10632377; doi:10.1038/s41467-023-42656-x)
Supplement: Supplementary file 3 — Reporting Summary [file 41467_2023_42656_MOESM3_ESM.pdf]

Corresponding author(s): Peter Scacheri

Last updated by author(s): Sep 7, 2023

Reporting Summary

Nature Portfolio wishes to improve the reproducibility of the work that we publish. This form provides structure for consistency and transparency in reporting. For further information on Nature Portfolio policies, see our Editorial Policies and the Editorial Policy Checklist.

Statistics

For all statistical analyses, confirm that the following items are present in the figure legend, table legend, main text, or Methods section.

n/a	Confirmed	
☐	☒	The exact sample size (*n*) for each experimental group/condition, given as a discrete number and unit of measurement
☐	☒	A statement on whether measurements were taken from distinct samples or whether the same sample was measured repeatedly
☐	☒	The statistical test(s) used AND whether they are one- or two-sided<br>*Only common tests should be described solely by name; describe more complex techniques in the Methods section.*
☒	☐	A description of all covariates tested
☒	☐	A description of any assumptions or corrections, such as tests of normality and adjustment for multiple comparisons
☐	☒	A full description of the statistical parameters including central tendency (e.g. means) or other basic estimates (e.g. regression coefficient) AND variation (e.g. standard deviation) or associated estimates of uncertainty (e.g. confidence intervals)
☐	☒	For null hypothesis testing, the test statistic (e.g. *F*, *t*, *r*) with confidence intervals, effect sizes, degrees of freedom and *P* value noted<br>*Give P values as exact values whenever suitable.*
☒	☐	For Bayesian analysis, information on the choice of priors and Markov chain Monte Carlo settings
☒	☐	For hierarchical and complex designs, identification of the appropriate level for tests and full reporting of outcomes
☐	☒	Estimates of effect sizes (e.g. Cohen's *d*, Pearson's *r*), indicating how they were calculated

Our web collection on statistics for biologists contains articles on many of the points above.

Software and code

Policy information about availability of computer code

Data collection	No software was used for data collection.

Data analysis	BWA-MEM version 0.7.12<br>Cutadapt version 1.9.1<br>Bedtools2 version 2.25.1<br>MACS version 2.1.2<br>Genrich version 0.5_dev<br>DeepTools version 3.2.0<br>R version 3.6.0<br>GimmeMotifs version 0.14.3<br>Crispresso2 version 2.0.32<br>HISAT2 version 2.1.0<br>Cell Ranger ATAC version 1.2<br>Seurat version 4.0.1<br>RSEM version 1.3.3<br>STAR version 2.5.3a<br><br>Custom code is available at https://github.com/wdpontius/temporal_chromatin_accessibility_osteo doi: 592872983

For manuscripts utilizing custom algorithms or software that are central to the research but not yet described in published literature, software must be made available to editors and reviewers. We strongly encourage code deposition in a community repository (e.g. GitHub). See the Nature Portfolio guidelines for submitting code & software for further information.

Data

Policy information about availability of data

All manuscripts must include a data availability statement. This statement should provide the following information, where applicable:

- Accession codes, unique identifiers, or web links for publicly available datasets
- A description of any restrictions on data availability
- For clinical datasets or third party data, please ensure that the statement adheres to our policy

Sequencing data generated for this manuscript can be found at the Gene Expression Omnibus (GEO) under the accession number GSE215765. The MNNG-HOS JQ1 RNA-seq data that was previously published is also available on GEO under the accession number GSE74230. DepMap data are available at https://depmap.org/portal/download/custom/. Human genome reference hg19 was used for sequencing alignment in this manuscript.

Human research participants

Policy information about studies involving human research participants and Sex and Gender in Research.

Reporting on sex and gender	*Use the terms sex (biological attribute) and gender (shaped by social and cultural circumstances) carefully in order to avoid confusing both terms. Indicate if findings apply to only one sex or gender; describe whether sex and gender were considered in study design whether sex and/or gender was determined based on self-reporting or assigned and methods used. Provide in the source data disaggregated sex and gender data where this information has been collected, and consent has been obtained for sharing of individual-level data; provide overall numbers in this Reporting Summary. Please state if this information has not been collected. Report sex- and gender-based analyses where performed, justify reasons for lack of sex- and gender-based analysis.*
Population characteristics	*Describe the covariate-relevant population characteristics of the human research participants (e.g. age, genotypic information, past and current diagnosis and treatment categories). If you filled out the behavioural & social sciences study design questions and have nothing to add here, write "See above."*
Recruitment	*Describe how participants were recruited. Outline any potential self-selection bias or other biases that may be present and how these are likely to impact results.*
Ethics oversight	*Identify the organization(s) that approved the study protocol.*

Note that full information on the approval of the study protocol must also be provided in the manuscript.

Field-specific reporting

Please select the one below that is the best fit for your research. If you are not sure, read the appropriate sections before making your selection.

☒ Life sciences ☐ Behavioural & social sciences ☐ Ecological, evolutionary & environmental sciences

For a reference copy of the document with all sections, see nature.com/documents/nr-reporting-summary-flat.pdf

Life sciences study design

All studies must disclose on these points even when the disclosure is negative.

Sample size	The sample size for animal experiments was based on our group's extensive experience with mouse models of metastasis. Each experiment was designed to minimize unnecessary animal use, optimize statistical power, and account for known variance in each model system.<br><br>Sample size for experiments not involving mice were determined by historical data, previous similar experiments, and expert judgment. While no formal sample size calculations were performed, the chosen sample sizes were based on ensuring adequate power for detecting meaningful differences or effects, given the variability observed in preliminary or past experiments. Sample sizes were sufficient to provide reliable and interpretable results, while also being mindful of resource constraints.
Data exclusions	No data were excluded.
Replication	Replicate experiments were performed as described in the methods. In vivo experiments were performed in triplicate, ex vivo experiments in triplicate, and in vitro experiments in quadruplicate or greater. All experiments showed similar results.
Randomization	Mice were randomly sorted into different cages at the beginning of experiments. Mice in the same cage received the same treatment (e.g. doxycycline water).
Blinding	No subjective measurements were used, thus researchers were not blinded to experimental groups.

Reporting for specific materials, systems and methods

We require information from authors about some types of materials, experimental systems and methods used in many studies. Here, indicate whether each material, system or method listed is relevant to your study. If you are not sure if a list item applies to your research, read the appropriate section before selecting a response.

Materials & experimental systems

n/a	Involved in the study
☐	☒ Antibodies
☐	☒ Eukaryotic cell lines
☒	☐ Palaeontology and archaeology
☐	☒ Animals and other organisms
☒	☐ Clinical data
☒	☐ Dual use research of concern

Methods

n/a	Involved in the study
☐	☒ ChIP-seq
☒	☐ Flow cytometry
☒	☐ MRI-based neuroimaging

Antibodies

Antibodies used	anti-KLF4 was purchased from R&D systems (AF3640) and used at 0.5 ug/mL<br>anti-cyclophilin B was purchased from abcam (ab16045) and used at 1:10,000 dilution<br>anti-H3K27ac was purchased from abcam (ab4729) and used at 8 ug per ChIP-seq<br><br>All secondary antibodies were used at a 1:10,000 dilution. Anti-goat and was purchased from Thermo Scientific, product # 31433, lot # RA2143996). Anti-rabbit was purchased from Thermo Scientific, product # 31460, lot # QG221919).
Validation	Antibody validation data are available on supplier websites.<br><br>anti-KLF4 was validated by western blot in HT-29 human colon adenocarcinoma cell line, SW480 human colorectal adenocarcinoma cell line, and HCT-116 human colorectal carcinoma cell line (https://www.rndsystems.com/products/human-klf4-antibody_af3640)<br><br>anti-cyclophilin B was validated by western blot in Wild-type HAP1 whole cell lysate, PPIB (Cyclophilin B) knockout HAP1 whole cell lysate, Jurkat whole cell lysate, and U87-MG whole cell lysate. Ab16045 was shown to specifically react with PPIB in wild-type HAP1 cells as signal was lost in PPIB knockout cells. (https://www.abcam.com/products/primary-antibodies/cyclophilin-b-antibody-ab16045.html)

Eukaryotic cell lines

Policy information about cell lines and Sex and Gender in Research

Cell line source(s)	MG63.3-GFP and 143b-HOS-GFP cell lines are originally from the lab of Dr. Chand Khanna. MG63.3-Cas9i-GFP was derived from MG63.3-GFP through lentiviral transduction, as described previously (doi: 10.1172/JCI127718)

Authentication	The authenticities of cell lines used in these studies have been confirmed previously by short tandem repeat (STR) profiling performed by the International Cell Line Authentication Committee.
Mycoplasma contamination	Routine testing confirming the absence of mycoplasma was performed using a custom PCR-based assay.
Commonly misidentified lines (See ICLAC register)	No commonly misidentified lines were used in this study.

Animals and other research organisms

Policy information about studies involving animals; ARRIVE guidelines recommended for reporting animal research, and Sex and Gender in Research

Laboratory animals	All mouse studies were performed with female NOD scid gamma mice (10-12 weeks) purchased from the CWRU Athymic Animal & Preclinical Therapeutics core facility. Mice were housed no more than five per cage in a room with a 12 h light/dark cycle with ad libitum access to water and rodent chow diet (Diet 7097, Harlan Teklad). Mice were kept at an ambient temperature of 23 °C and humidity of 40-60%.
Wild animals	No wild animals were used in this study.
Reporting on sex	Sex was not considered in the study design.
Field-collected samples	No field-collected samples were used in this study.
Ethics oversight	All mouse experiments were approved under IACUC protocol 2014-0156. Mice were housed in ultraclean facilities in accordance with protocols approved by the CWRU Institutional Animal Care and Use Committee.

Note that full information on the approval of the study protocol must also be provided in the manuscript.

ChIP-seq

Data deposition

- [x] Confirm that both raw and final processed data have been deposited in a public database such as GEO.
- [x] Confirm that you have deposited or provided access to graph files (e.g. BED files) for the called peaks.

Data access links *May remain private before publication.*	Raw and processed data are available at the accession number GSE215765.
Files in database submission	H3K27ac ChIP-seq on non-target and sgKLF4 transduced MG63.3-Cas9i-GFP cells.
Genome browser session (e.g. UCSC)	No longer applicable.

Methodology

Replicates	ChIP-seq experiments were not performed with replicates.
Sequencing depth	Libraries were sequenced paired-end with 150bp reads.
Antibodies	anti-H3K27ac was purchased from abcam (ab4729)
Peak calling parameters	Data were aligned to hg19 using BWA-MEM with default parameters in paired-end mode. Output SAM files were converted to binary format, sorted, indexed, and removed of PCR duplicates using SAMtools v1.10. Peaks were called using MACS v2.1.2 with the --broad flag set.
Data quality	BigWigs were visualized on the Integrative Genomics Viewer (IGV) to assess pronounced track irregulatrities or low signal-to-noise ratio.
Software	Custom pipeline for analyzing ChIP-seq can be found at: https://github.com/scacherilab/ChIP-Seq_pipeline
